# Cytokine Profiles in Malawian Children Presenting with Uncomplicated Malaria, Severe Malarial Anemia, and Cerebral Malaria

**DOI:** 10.1128/CVI.00533-16

**Published:** 2017-04-05

**Authors:** Wilson L. Mandala, Chisomo L. Msefula, Esther N. Gondwe, Mark T. Drayson, Malcolm E. Molyneux, Calman A. MacLennan

**Affiliations:** a Malawi-Liverpool-Wellcome Trust Clinical Research Programme, College of Medicine, University of Malawi, Blantyre, Malawi; b Department of Basic Medical Sciences, College of Medicine, University of Malawi, Blantyre, Malawi; c Liverpool School of Tropical Medicine, Liverpool, United Kingdom; d Department of Microbiology, College of Medicine, University of Malawi, Blantyre, Malawi; e Institute of Immunology and Immunotherapy, College of Medicine and Dental Sciences, University of Birmingham, Birmingham, United Kingdom; f Department of Medicine, College of Medicine, University of Malawi, Blantyre, Malawi; g The Jenner Institute, Nuffield Department of Medicine, University of Oxford, Oxford, United Kingdom; University of Florida

**Keywords:** cytokines, malaria

## Abstract

Proinflammatory cytokines are involved in clearance of Plasmodium falciparum, and very high levels of these cytokines have been implicated in the pathogenesis of severe malaria. In order to determine how cytokines vary with disease severity and syndrome, we enrolled Malawian children presenting with cerebral malaria (CM), severe malarial anemia (SMA), and uncomplicated malaria (UCM) and healthy controls. We analyzed serum cytokine concentrations in acute infection and in convalescence. With the exception of interleukin 5 (IL-5), cytokine concentrations were highest in acute CM, followed by SMA, and were only mildly elevated in UCM. Cytokine concentrations had fallen to control levels when remeasured at 1 month of convalescence in all three clinical malaria groups. Ratios of IL-10 to tumor necrosis factor alpha (TNF-α) and of IL-10 to IL-6 followed a similar pattern. Children presenting with acute CM had significantly higher concentrations of TNF-α (*P* < 0.001), interferon gamma (IFN-γ) (*P* = 0.0019), IL-2 (*P* = 0.0004), IL-6 (*P* < 0.001), IL-8 (*P* < 0.001), and IL-10 (*P* < 0.001) in sera than healthy controls. Patients with acute CM had significantly higher concentrations of IL-6 (*P* < 0.001) and IL-10 (*P* = 0.0003) than those presenting with acute SMA. Our findings are consistent with the concept that high levels of proinflammatory cytokines, despite high levels of the anti-inflammatory cytokine IL-10, could contribute to the pathogenesis of CM.

## INTRODUCTION

Nearly 214 million clinical episodes of malaria were reported in 2015, leading to 438,000 deaths, the majority of which were among African children and attributable to Plasmodium falciparum malaria (1). Clinical P. falciparum malaria presents either as uncomplicated malaria (UCM) or as one of the following severe forms of the disease: cerebral malaria (CM), severe malarial anemia (SMA), metabolic acidosis (MA), or respiratory distress (RD) and other complications, including some overlap syndromes (1, 2).

Immunity to malaria is both humoral and cell mediated and involves various mechanisms (3). Antibodies that develop through exposure to P. falciparum play a role (3), and the involvement of different lymphocyte subsets has been implicated in both protection against, and pathogenesis of, malaria (4–6).

Cytokines are regulatory proteins or glycoproteins secreted by white blood cells and various other cells in response to a number of stimuli (7). “Cytokine” is a general term, but cytokines have more-specific names depending on the type of cells that produce them and on the functions that they perform, such that lymphokines are produced by lymphocytes and monokines by monocytes and macrophages (8). Lymphokines such as interferon gamma (IFN-γ) and interleukin 4 (IL-4) stimulate B cells to produce antibodies and attract and activate immune cells such as macrophages and other lymphocytes at sites of infection (8–11). In contrast, monokines such as tumor necrosis factor alpha (TNF-α), IL-1, IL-6, and IL-8 play roles that are inflammatory in nature and also attract neutrophils by chemotaxis (9, 10). However, it is clear now that the majority of cytokines can be produced by a range of different cell types, questioning the apparent specificity of “lymphokine” and “monokine.”

Cytokines can also be grouped based on the T cells that produce them when the T cells are stimulated to differentiate. T helper 1 (Th1) cells are known to produce large quantities of IFN-γ, induce delayed hypersensitivity reactions, and activate macrophages and are crucial for the defense against intracellular pathogens (11, 12), whereas Th17 cells produce IL-17, IL-21, and IL-22 (11). Th2 cells produce IL-4 and are important in inducing IgE production, recruiting eosinophils to sites of inflammation and helping clear parasitic infections (8, 11).

When categorized based on their effect on inflammation, cytokines can be termed proinflammatory, with the cytokines IL-1, TNF-α, IFN-γ, IL-12, and IL-18 included in this group, while cytokines such as IL-4, IL-10, IL-13, and transforming growth factor beta (TGF-β) are referred to as anti-inflammatory cytokines (12, 13). Proinflammatory cytokines are produced by a multiplicity of cells, including lymphocytes, monocytes, macrophages, fibroblasts, neutrophils, endothelial cells, and mast cells, and are known to be involved in clearing the initial parasitemia in the early stages of P. falciparum infection (7, 14, 15). Proinflammatory cytokines such as TNF-α (16), IFN-γ, IL-6, and IL-1 (17, 18), when produced in an unregulated manner, have been implicated in the pathogenesis of cerebral malaria (19) and correlate with disease severity and death (20).

In contrast, anti-inflammatory cytokines such as IL-10 have been shown to downregulate the proinflammatory cytokines (15, 21). Experiments in which IL-10 was administered in mouse models of malaria resulted in a lower production of TNF-α and a lower incidence of experimental cerebral malaria (ECM) (22, 23), leading some to hypothesize that IL-10 counteracts the potentially pathological host proinflammatory response to malaria (14).

Inflammatory cytokines also play an important role in the pathogenesis of SMA, with high levels of TNF-α but low levels of IL-10 (24) being associated with SMA in areas of high malaria endemicity (24, 25). IL-12 has been shown to be involved in protective immunity against malaria by regulating IFN-γ, TNF-α, and nitric oxide responses in experimental studies (26) and enhancing erythropoiesis in Plasmodium chabaudi-infected susceptible mice (27).

Although cytokines may act on the same cells that secrete them (autocrine action), on cells within close proximity (paracrine action), or in some cases on distant cells (endocrine action) (8), *in vitro* assays can only either measure the proportions of cytokine-producing cells by intracellular cytokine staining or quantify cytokine concentrations in serum or plasma samples extracted from stimulated or unstimulated venous blood samples using commercially available enzyme immunoassays (28). We analyzed serum samples from children presenting with different clinical presentations of malaria during acute infection and in convalescence, together with samples from healthy children, in order to determine the concentrations of different cytokines.

## RESULTS

### Characteristics of study children.

The number of participants recruited in each group and their demographic and hematological characteristics have been published previously (6) and are presented in Table 1. Briefly, consent was obtained for 188 children aged 5 to 84 months to participate in the study. Blood samples from 33 children were excluded for the following reasons: HIV infection (*n* = 14), malaria parasites in the blood of control subjects (*n* = 14), Blantyre coma score (BCS) greater than 2 at 4 h postadmission in children with suspected CM (*n* = 4), and hemoglobin below 5 g/dl in one child with CM. Five children (four with CM and one with SMA) died days after therapy had been administered.

**TABLE 1:** Demographic and clinical details of study participants^*a*^

Characteristic	Controls	Cerebral malaria	Severe malarial anemia	Uncomplicated malaria
No. of participants	42	29	30	54
No. who died after recruitment		4	1	0
No. reviewed in convalescence		18	21	34
Sex (M:F)	29:13	10:19	19:11	38:16
Age (mo)	20 (5–76)	30 (5–84)	23 (5–38)	27 (6–58)
No. of parasites/μl blood	0	41,800 (900–517,000)	3,500 (20–296,000)	52,300 (460–768,000)
Blantyre coma score	5	1 (0–2)	5	5
Hemoglobin concn (g/dl)	11.2 (7.0–14.1)	7.7 (5.3–12.5)	3.9 (2.4–4.9)	9.3 (5.0–13.0)

a Subjects were children with cerebral malaria, severe malarial anemia, and uncomplicated malaria presenting to the Pediatric Accident and Emergency Clinic at Queen Elizabeth Central Hospital in Blantyre, Malawi. Control subjects were children admitted for elective surgical procedures who were medically healthy. Values are medians (ranges). These participants' details have been published previously (6).

### Cytokine concentrations during acute infection and in convalescence.

**FIG 1:**
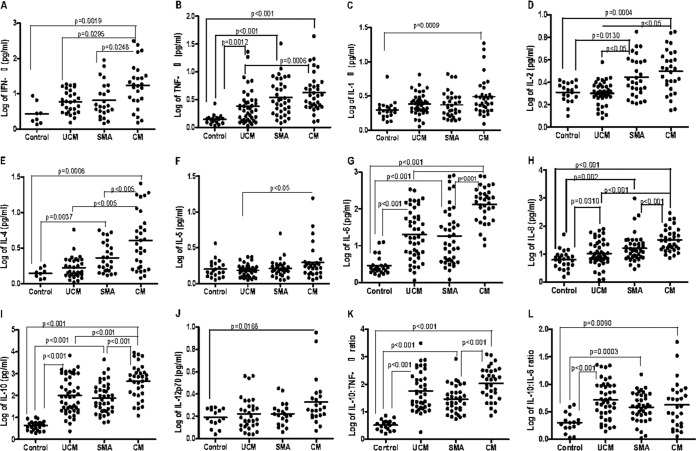
(A to J) Plots of log-transformed concentrations (picograms per milliliter) of different cytokines (IFN-γ, TNF-α, IL-1β, IL-2, IL-4, IL-5, IL-6, IL-8, IL-10, and IL-12p70) in serum samples collected from healthy controls (Control) and from patients with acute uncomplicated malaria (UCM), acute severe malarial anemia (SMA), and acute cerebral malaria (CM). (K and L) Plots of the ratios of log-transformed IL-10 to TNF-α and IL-10 to IL-6, respectively, during acute infection, showing medians and 10th and 90th percentiles.

**FIG 2:**
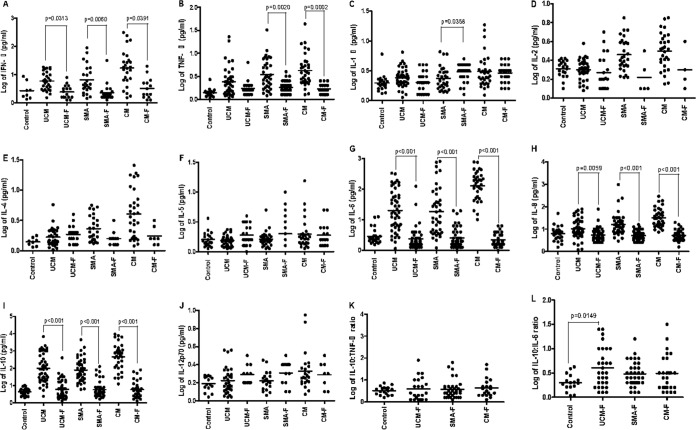
(A to J) Plots of log-transformed concentrations (in picograms per milliliter) of different cytokines (IFN-γ, TNF-α, IL-1β, IL-2, IL-4, IL-5, IL-6, IL-8, IL-10, and IL-12p70) in serum samples collected from healthy controls (Control) and from patients with convalescent uncomplicated malaria (UCM-F), severe malarial anemia (SMA-F), and cerebral malaria (CM-F). (K and L) Plots of the ratios of log-transformed IL-10 to TNF-α and IL-10 to IL-6, respectively, during convalescence, showing medians and 10th and 90th percentiles.

The median concentration of IFN-γ (Fig. 1A; see also Table S1 in the supplemental material) was significantly (*P* = 0.0019) higher in acute CM cases (17.3 pg/ml) than in controls (2.32 pg/ml) and in acute SMA (*P* = 0.0248) and acute UCM (*P* = 0.0295) cases, and these levels then decreased significantly (*P* < 0.001) in convalescence (Fig. 2A). TNF-α levels during acute disease were higher in all types of clinical malaria than in controls (Fig. 1B), with significant (*P* = 0.0313 for UCM, *P* = 0.0060 for SMA, and *P* = 0.0391 for CM) differences observed between CM patients (median, 3.76 pg/ml) and controls (median, 1.41 pg/ml) and between SMA patients (median, 2.95 pg/ml) and controls. UCM patients also had significantly (*P* = 0.0012) higher TNF-α levels (2.12 pg/ml) than controls (1.41 pg/ml) but significantly (*P* = 0.0006) lower levels than CM cases (median, 3.76 pg/ml). TNF-α levels decreased significantly in convalescence (Fig. 2B) for both CM (median, 3.76 pg/ml, falling to 1.69 pg/ml; *P* = 0.0002) and SMA (median, 2.95 pg/ml, falling to 1.80 pg/ml; *P* = 0.0020).

Acute CM patients had significantly (*P* = 0.0009) higher median concentrations of IL-1β (2.48 pg/ml) than controls (median, 1.89 pg/ml) during acute infection (Fig. 1C), and surprisingly, the level in the CM group remained elevated (median, 3.09 pg/ml) in convalescence (Fig. 2C). SMA patients had significantly (*P* = 0.0358) higher median concentrations of IL-1β during convalescence (median, 3.34 pg/ml) than during acute disease (median, 2.21 pg/ml) (Fig. 2C).

Both CM patients (median, 3.34 pg/ml) and SMA patients (median, 2.55 pg/ml) had significantly higher (*P* = 0.0004 for CM and *P* = 0.0130 for SMA) levels of IL-2 in acute disease than controls (2.12 pg/ml) (Fig. 1D). Levels in both CM and SMA were significantly higher (*P* < 0.05) than levels in acute UCM (2.02 pg/ml). IL-2 levels in convalescence in all malaria types were similar to those of controls (medians, 1.60 pg/ml for UCM, 1.30 pg/ml for SMA, 1.65 pg/ml for CM) (Fig. 2D).

On admission, CM (median, 3.62 pg/ml) and SMA (median, 2.03 pg/ml) groups had significantly (*P* = 0.0006 for CM and *P* = 0.0037 for SMA) higher concentrations of IL-4 than controls (median, 1.41 pg/ml) (Fig. 1E), with levels in the disease groups decreasing in convalescence (Fig. 2E). IL-4 levels in acute CM were significantly (*P* < 0.005) higher than those in acute SMA and UCM (median, 1.49 pg/ml). Concentrations of IL-5 in acute infection of all three types of malaria were similar to those of healthy controls (Fig. 1F), although the levels of IL-5 in acute CM (median, 1.74 pg/ml) were significantly higher (*P* < 0.05) than the levels in UCM (median, 1.54 pg/ml). IL-5 levels in all three malaria types were similar in acute infection and in convalescence (Fig. 2F).

IL-6 levels in all three malaria types (medians, 17.31 pg/ml for UCM, 12.20 pg/ml for SMA, and 156.3 pg/ml for CM) were significantly (*P* < 0.001) higher during acute disease than in controls (2.37 pg/ml) (Fig. 1G). Among the three malaria types, CM patients had the highest IL-6 levels and the differences between the levels in acute SMA and CM and between acute UCM and CM were significant (*P* < 0.001). All three malaria types had significantly (*P* < 0.001) lower IL-6 levels (medians, 1.90 pg/ml for UCM, 2.09 pg/ml for SMA, and 1.87 pg/ml for CM) in convalescence (Fig. 2G) than in acute infection.

During acute illness (Fig. 1H), IL-8 levels were higher in all malaria types (medians, 8.47 pg/ml for UCM, *P* = 0.031; 13.03 pg/ml for SMA, *P* = 0.002; and 29.71 pg/ml for CM, *P* < 0.001) than in controls (median, 6.55 pg/ml). IL-8 concentrations in CM patients were significantly (*P* < 0.001) higher than in both SMA and UCM patients. In convalescence, IL-8 levels had significantly decreased in all three malaria types (*P* = 0.0059 for UCM and *P* < 0.001 for SMA and CM) (Fig. 2H).

During acute illness, median IL-10 levels were significantly (*P* < 0.001 for UCM, SMA, and CM) higher in children presenting with each of the malaria syndromes than in controls (Fig. 1I). Similar to the trend for IL-8, levels of IL-10 in acute SMA and UCM patients were significantly (*P* < 0.001) lower than in acute CM. IL-10 levels in all malaria groups were significantly (*P* < 0.001) lower in convalescence (medians, 4.13 pg/ml for UCM, 4.50 pg/ml for SMA, and 4.64 pg/ml for CM) than in acute disease (Fig. 2I). Patients presenting with acute CM had significantly (*P* = 0.0168) higher levels of IL-12p70 (median, 2.09 pg/ml) than controls (median, 1.52 pg/ml) (Fig. 1J). Levels of IL-12p70 were similar in acute infection and convalescence (Fig. 2J).

IL-12 is the main driver of the IFN-γ response in the T helper 1 pathway, and so, perhaps surprisingly, IL-12p70 was significantly elevated only in CM compared with controls, but only to a modest degree (medians, 2.13 pg/ml and 1.52 pg/ml; *P* = 0.0168).

**FIG 3:**
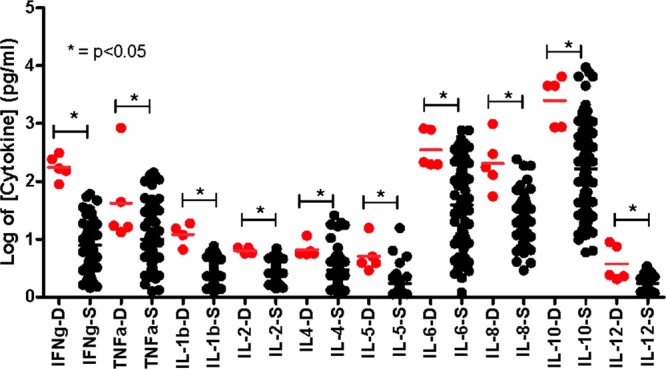
Cytokine levels in children with CM and SMA who died or who survived. Plot of log-transformed concentrations (in picograms per milliliter) of different cytokines (IFN-γ, TNF-α, IL-1β, IL-2, IL-4, IL-5, IL-6, IL-8, IL-10, and IL-12p70) in serum samples collected from five children who died (D, red dots) and those children who survived (S, black dots) after presenting with acute CM and SMA, showing medians and 10th and 90th percentiles.

CM patients (*n* = 4) and one SMA patient who had died had significantly (*P* < 0.05) higher levels of all cytokines (Fig. 3; Table 2) during acute illness than those who survived (*n* = 25).

**TABLE 2:** Median concentrations of different cytokines in serum samples collected from children

Cytokine	Median concn (pg/ml) in serum samples from:		*P* value
	Dead children (*n* = 5)^*a*^	Surviving children (*n* = 25)^*b*^	
IFN-γ	2.22	0.94	0.0030
TNF-α	1.23	0.76	0.0543
IL-1β	1.125	0.385	0.0010
IL-2	0.805	0.45	0.0012
IL-4	0.75	0.425	0.0065
IL-5	0.59	0.22	0.0009
IL-6	2.33	1.84	0.0061
IL-8	2.24	1.24	0.0007
IL-10	3.65	2.34	0.0039
IL-12p70	0.38	0.22	0.0035

a Four children had CM, and 1 child had SMA.

b Children who survived after presenting with acute CM (*n* = 11) and SMA (*n* = 14).

### Comparison of IL-10/TNF-α and IL-10/IL-6 ratios between different groups.

The IL-10-to-TNF-α ratio was significantly (*P* < 0.001) higher in acute UCM, SMA, and CM (medians, 43.25, 25.16, and 140.2, respectively) patients than in controls (median, 3.47), while the ratio in acute SMA was significantly (*P* < 0.001) lower than the ratio in acute CM (Fig. 1K). The IL-10-to-TNF-α ratios for all three malaria types (medians, 2.10 for UCM, 2.60 for SMA, and 3.65 for CM) in convalescence were similar to ratios in controls (median, 1.63) (Fig. 2K).

The IL-10-to-IL-6 ratios in acute UCM, SMA, and CM (medians, 4.58, 3.92, and 2.93, respectively) were significantly (*P* < 0.001 for UCM, *P* = 0.0003 for SMA, and *P* = 0.0090 for CM) higher than the ratio in controls (median, 1.63) (Fig. 1L and Table 2). During convalescence, UCM (median, 2.20) still had significantly (*P* = 0.0149) higher IL-10-to-IL-6 ratios than controls (median, 1.63) but SMA (median, 2.20) and CM (median, 2.20) ratios were just as low as those of controls (Fig. 2L; Table S1).

## DISCUSSION

Cytokine production by different cell types in response to foreign antigen is one of the defense mechanisms that characterize cellular immunity and can drive both normal and pathological immune responses (7). Previous studies have shown that when proinflammatory cytokines (produced by a variety of cells, including Th1 cells and macrophages) such as TNF-α (16), IFN-γ, IL-6, and IL-1 (17, 18) are produced in an unregulated manner, they contribute to the pathogenesis of cerebral malaria (19) and to disease severity and death (20). In contrast, anti-inflammatory cytokines (produced by cells that include monocytes and Th2 cells) such as IL-10 and IL-13 have been shown to downregulate the production of proinflammatory cytokines (15, 21) and to reduce the incidence of experimental cerebral malaria (ECM) in mouse models (22).

We analyzed concentrations of serum cytokines in Malawian children presenting with CM, SMA, and UCM in acute illness and in convalescence and compared these levels with those in healthy controls (Table S1; Table 2). We found that both proinflammatory (TNF-α, IFN-γ, IL-1, IL-6) and anti-inflammatory (mainly IL-10) cytokine concentrations were markedly elevated over control levels in Malawian children presenting with CM, moderately raised in SMA patients, and minimally but significantly increased in those children presenting with UCM. In all patient groups, cytokine concentrations decreased to control levels in convalescence. A similar trend was observed for IL-10-to-TNF-α and IL-10-to-IL-6 ratios. These results indicate that acute malaria, regardless of severity, is characterized by higher-than-normal levels of a broad range of, but not all, cytokines, whether in the “Th1 group” (IFN-γ, TNF-α, and IL-1) or the “Th2 group” (IL-4, IL-6, and IL-10). These high levels decrease significantly in convalescence.

In line with our findings, most studies that have determined cytokine concentrations in Plasmodium malaria, both in mouse models (22, 26, 27) and in humans (12, 14, 17, 21, 23, 29–41), have reported highly elevated cytokine concentrations in symptomatic malaria of all clinical varieties. Although murine studies provide some insight into malaria-related cytokine perturbations, data from humans with various malaria syndromes are essential for understanding the pathogenesis of human disease.

Among the various studies that have investigated cytokine perturbation in P. falciparum malaria in other countries (12, 29–41), few have compared the levels in the different clinical types of malaria, namely, UCM, SMA, and CM (12, 14). TNF-α, IFN-γ, IL-1, IL-6, IL-8, and IL-10 have been found in increased levels in patients with severe malaria compared to healthy controls (14, 25), decreasing in convalescence to control levels, but in these studies the clinical syndromes of severe malaria were not fully described. Cytokine data from children presenting with strictly defined UCM, SMA, and CM and a month in convalescence in this study provide additional valuable information.

Interestingly, there are some apparent paradoxes between the cytokine concentrations reported in this paper and the monocyte intracellular cytokine staining (42) and immunophenotyping (6) findings that we have reported previously for the same study participants. We observed decreased IL-6 and TNF-α production by monocytes in children with different forms of malaria (42). This indicates that the elevated levels of these cytokines in serum in the current report are produced by cells other than monocytes (or macrophages), most likely T cells and NK cells. Moreover, we reported panlymphopenia among children with cerebral malaria and uncomplicated malaria. Therefore, elevated cytokine production by lymphocytes in these groups would have to come either from, counterintuitively, a reduced number of peripheral blood lymphocytes or from lymphocytes retained in secondary lymphoid tissues or sequestered in other vascular structures.

Not all malaria-infected children with high levels of Th1 proinflammatory cytokines, such as TNF-α, develop severe malaria (14), suggesting that the cytokine network as a whole, rather than a single cytokine, may contribute in different ways to severe disease (12, 25). Thus, severe P. falciparum malaria could be associated with an inadequate negative-feedback response by Th2 anti-inflammatory cytokines such as IL-10. The timing of IL-10 production is likely to be important in determining the effectiveness of IL-10 as an anti-inflammatory cytokine, with *in vitro* studies showing that TNF-α, IL-6, and IL-1β are produced within 2 to 4 h of stimulation while IL-10 is first detected after 8 h, supporting the concept that IL-10 counterregulates the proinflammatory response to P. falciparum (12). The *in vitro* observations that IL-10 was detected 7 h after activation of monocytes with lipopolysaccharide (LPS) and that maximal IL-10 levels were observed only after 24 to 48 h of stimulation with LPS (43) are consistent with this concept.

Since IL-10 serves to regulate both the production and functions of TNF-α and IL-6 (19, 43), it has been suggested that children with a low IL-10-to-TNF-α ratio may be more likely to develop severe malaria than children with a higher ratio (14). In a study from Kenya (25), children with severe malaria (the type of severe malaria was not specified) had higher IL-10-to-TNF-α ratios than did children presenting with mild disease. Here we found that Malawian children presenting with all forms of malaria had high IL-10-to-TNF-α and IL-10-to-IL-6 ratios, so high levels of TNF-α and IL-6 in CM could not be attributed to a lack of IL-10 response. Nevertheless, it is apparent that the IL-10 response observed in CM was unable to prevent these high proinflammatory cytokine levels, since higher levels of IL-6 and TNF-α in malaria patients who died than in those who survived, as found previously in adults in Vietnam (19), suggest that uncontrolled levels of these cytokines may have contributed to the demise of these children. In a separate study in Malawian children, those presenting with severe malaria had higher levels of IL-6 and TNF-α than did those presenting with UCM, although severe malaria was not further subcategorized (29).

Overall, the observation that higher levels of the proinflammatory/Th1 cytokines were found in CM than in SMA is consistent with the concept that CM results from an immunopathological response in which the production of proinflammatory cytokines is poorly regulated (19, 43). Other investigators have argued that early, as opposed to late, production of IFN-γ and TNF-α correlates well with protection, since when these are produced early, overproduction can more easily be kept in check by the presence of anti-inflammatory cytokines such as IL-10 (30).

The present study was limited in that the analyzed blood samples were collected only at two time points, one at the acute phase and one in convalescence, that were roughly 30 days apart. It would be informative to conduct a longitudinal study recruiting children that present with different forms of malaria who are then monitored closely to provide a time course curve for these cytokines, as has been done before with blood samples from South African adults (37), although these were monitored for only 5 days.

An unavoidable limitation of clinical studies of natural infection is that we do not know the point in time at which plasmodial sporozoites are first inoculated by the mosquito, nor do we know the time when merozoites first emerge from the liver to invade erythrocytes. Although we have reported on the proportion of cytokine-producing monocytes from each of these three malaria groups (42), inclusion of an intracellular cytokine analysis for other cytokine-producing cells could enable the identification of the main producers of the cytokines present in the corresponding serum/plasma. Lastly, although this study analyzed serum samples for concentrations of some cytokines, analysis of additional cytokines that are suspected to play some roles in malaria immunity (40, 41) as well as concentrations of chemokines such as RANTES and IP-10, which have been shown to vary with malaria severity (40), would provide additional insight into their separate and/or synergistic roles in malaria. Subsequent studies should combine analysis of serum or plasma samples for cytokines with intracellular cytokine staining in samples collected from children presenting with different forms of clinical malaria.

We have shown that just as different clinical malaria syndromes are characterized by diverse perturbations of leukocyte and lymphocyte subsets (6), they are also characterized by altered cytokine patterns. While in acute CM there are a transient panlymphopenia (6) and the lowest proportion of IL-6- and TNF-α-producing monocytes (42), there is a paradoxical concomitant elevation of circulating cytokine levels, with all perturbations normalizing in convalescence (6, 42). Many studies of cytokine levels in malaria have been published, yielding generally similar findings. In this paper, however, we bring together various malarial syndromes, different classes of cytokines, and admission and convalescent time points in the same group of children, in whom circulating leukocyte counts and lymphocyte subsets have already been quantitated. The question as to why the cells that might have been expected to increase in association with their secretory product actually decrease during acute illness (6) could be addressed by the hypothesis that the secretory cells get sequestered in secondary lymphoid tissue during acute disease, whereas the observation of high cytokine levels in acute disease but low proportion of cytokine-producing monocytes (42) could be explained by the hypothesis that the monocytes responsible for producing the observed high cytokine levels are anergic to further stimulation during the phase of acute disease when a venous sample is collected.

Our findings support the suggestion that cytokines, particularly in CM, may promote the transient sequestration of lymphocytes in secondary lymphoid tissue, potentially causing the observed paradoxical lymphopenia (6) by contributing to the upregulation of CD69 that is recognized in CM (44). The differences in cytokine levels between CM and other malarial syndromes may reflect the severity of the disease (CM has the highest case fatality rate among these syndromes) and/or the parasite burden, which, in most studies that include these three syndromes, is greatest in CM (2).

With developing technologies for bedside diagnosis, patterns of circulating cytokine concentrations may in due time contribute to the rapid differentiation between malaria and other causes of fever. For this possibility to be realized, increased amounts of data on immunological and biochemical parameters, including cytokine levels, will need to be gathered from clinically well-characterized patients, so that new tests can be evaluated for their practical usefulness.

## MATERIALS AND METHODS

### Study area and study population.

The study was conducted within the Malawi-Liverpool-Wellcome Trust Clinical Research Programme and Department of Pediatrics, College of Medicine, University of Malawi, and Blantyre Malaria Project. Participants were children admitted with acute malaria to Queen Elizabeth Central Hospital (QECH) and medically healthy children attending surgical outpatient clinics at QECH and Beit Cure International Hospital, both in Blantyre, Malawi. Demographic and clinical features of the participants have been reported previously (6). In brief, children were enrolled during the rainy season (November 2005 to April 2006) after informed consent was obtained from the parent or guardian. Each child was examined by a research nurse and clinical officer, baseline demographic data were recorded, and a venous blood sample was collected. Criteria defining clinical malaria were fever, a clinical syndrome compatible with malaria without any apparent alternative cause, and a thick blood film positive for Plasmodium falciparum asexual parasites on microscopy. Children were assessed for level of consciousness using the Blantyre coma score (BCS) on admission and at 2- to 4-hour intervals during intensive clinical care. Over 40 children were prospectively enrolled into each of the four clinical groups defined by diagnoses of cerebral malaria (CM), severe malarial anemia (SMA), or uncomplicated malaria (UCM) or healthy controls.

Children with CM had a BCS of 2 or less at admission and 4 h later, while children in all other groups had a score of 5 at both times (Table 1). Children with SMA had a blood hemoglobin concentration of 5 g/dl or less, and all other children had a hemoglobin concentration of >5 g/dl. Children who tested positive for HIV infection were excluded from the study and referred to the antiretroviral therapy clinic. Children who presented with UCM or SMA were treated with a standard regimen of sulfadoxine-pyrimethamine (SP), which was the first-line treatment for malaria in Malawi at the time the study was conducted. In contrast, children presenting with CM were treated with intramuscular (i.m.) quinine as recommended for CM patients at that time. Study participants in the UCM, SMA, and CM groups were seen again approximately 30 days after treatment (convalescence or follow-up [F] visit), at which time a second blood sample was collected.

### Malaria microscopy.

Thick and thin films were prepared for determining the density of malaria parasitemia. Preparation and reading of malaria slides were performed in accordance with standard WHO procedures (45). Briefly, two blood slides were prepared from each participant's blood sample. Each slide had a measured volume of 6 μl of blood for the thick film and 2 μl for the thin film. A 3% working stock of Giemsa stain was prepared using a principal Giemsa-staining stock solution and Giemsa buffer prepared from buffer tablets. Thin and thick blood smears were stained with Giemsa after fixing the thin smear with absolute methanol. The stained slides were read by two competent, independent malaria microscopists. The entire smear was first screened at a low magnification (10× and 40× objective lenses) to detect suitable fields with even distribution of white blood cells (WBC) (10 to 20 WBC/field). Smears were then examined using a 100× oil immersion lens. At least 100 low-power fields were examined before a thick smear was declared negative. A blood slide was declared positive when a concordant result was produced by the microscopists. P. falciparum parasites were counted per 200 or 500 leukocytes, in order to estimate the parasite density per microliter of blood. Discordant results were resolved by a third reading of the films. Thin films were examined to confirm the species of the infecting Plasmodium.

### HIV and malaria tests.

HIV testing was performed using two rapid tests, Determine (Abbott Laboratories, Tokyo, Japan) and UniGold (Trinity Biotech, Dublin, Ireland). Discordant results and positive results in children under 18 months were confirmed by PCR as previously described (46).

### Serum collection and preservation.

Whole-blood samples from study participants were collected on admission (prior to administering antimalarial therapy) and 1 month after treatment as previously described (6). An aliquot of the blood sample was collected in a plain tube and allowed to coagulate with serum separation by centrifugation. Serum was divided into aliquots and preserved at −80°C until required for cytokine analysis.

### Cytokine analysis.

Concentrations of various cytokines were determined using Becton Dickinson (BD) cytokine bead array (CBA) kits. Sera were thawed and centrifuged at maximum speed for 10 min to remove fibrin deposits. A 25-μl volume of each sample was mixed with 25 μl of the capture bead mixture and then with 25 μl of detection reagent. Subsequent steps were performed according to the manufacturer's instructions (BD CBA Instruction Manuals, 2006). The kit sensitivity (minimum detectable concentration) limits for the various cytokines are provided in Table S1.

### Statistical analysis.

Statistical tests were performed using GraphPad Prism version 6.01 for Windows (GraphPad Software, San Diego CA, USA). The Kruskall-Wallis test was used to compare the medians of the different cytokine concentrations (in picograms per milliliter) and ratios in different clinical groups. Between-group comparisons of cytokine concentrations for the four groups (controls, UCM, SMA, and CM) were assessed with Bonferroni's multiple-comparison test, and *P* values of <0.0125 were considered statistically significant. The Wilcoxon matched-pair test was used to determine the statistical significance of the differences in concentrations and ratios observed during acute infection and in convalescence for each clinical syndrome of malaria, and *P* values of <0.05 were considered statistically significant.

### Ethical approval.

The study was approved by the College of Medicine Research and Ethics Committee, University of Malawi, and Ethics Committee of the Liverpool School of Tropical Medicine, UK.

## Supplementary Material

Supplemental material
