# Supplementary material for: Cytokine Profiles in Malawian Children Presenting with Uncomplicated Malaria, Severe Malarial Anemia, and Cerebral Malaria
Source: Clin Vaccine Immunol. 2017 Apr 5;24(4):e00533-16. doi: 10.1128/CVI.00533-16 (PMC5382826; doi:10.1128/CVI.00533-16)
Supplement: Supplemental material [file supp_24_4_e00533-16__index.html]

Supplemental material 

# Cytokine Profiles in Malawian Children Presenting with Uncomplicated Malaria, Severe Malarial Anemia, and Cerebral Malaria

## Supplemental material

- Supplemental file 1 -

  Table S1. Cytokine concentrations (pg/ml) and ratios in controls and patients with different malaria types during acute infection and in convalescence.

  PDF, 38K
