# Supplementary material for: Cytokine Profiles in Malawian Children Presenting with Uncomplicated Malaria, Severe Malarial Anemia, and Cerebral Malaria
Source: Clin Vaccine Immunol. 2017 Apr 5;24(4):e00533-16. doi: 10.1128/CVI.00533-16 (PMC5382826; doi:10.1128/CVI.00533-16)
Supplement: Supplemental material [file CVI.00533-16_zcd999095458s1.pdf]

Table S1: Cytokine concentrations (pg/ml) and ratios in controls and patients of different malaria types during acute infection and in convalescence.

Cytokine (limit of detection)	Medians (10th and 90th percentiles)						
	Control	UCM		SMA		CM	
	(n = 42)	Acute (n = 54)	Follow-up (n = 34)	Acute (n = 30)	Follow-up (n = 21)	Acute (n = 29)	Follow-up (n = 18)
IFN-γ (7.2)	2.32 (1.35- 8.66)	5.86 (2.42- 17.07)	1.91 (1.10- 5.58)	4.43 (1.74- 36.19)	1.80 (1.22- 3.35)	17.28 (2.02- 55.03)	1.89 (1.13- 5.76)
TNF-α (2.8)	1.41 (1.12- 1.76)	2.12 (1.22- 6.35)	1.50 (1.26- 2.21)	2.95 (1.42- 8.28)	1.80 (1.19- 2.63)	3.76 (1.90- 13.41)	1.69 (1.18- 2.44)
IL-1β (7.2)	1.89 (1.37- 2.50)	2.46 (1.45- 4.09)	2.00 (1.30- 4.22)	2.21 (1.44- 5.40)	3.34 (1.90- 4.03)	2.48 (1.68- 5.08)	3.09 (1.65- 4.66)
IL-2 (2.6)	2.12 (1.46- 2.60)	2.02 (1.37- 2.67)	1.60 (1.20- 3.70)	2.55 (1.67- 5.20)	1.30 (1.20- 3.20)	3.34 (1.70- 5.70)	1.65 (1.20- 3.60)
IL-4 (2.6)	1.41 (1.14- 1.78)	1.49 (1.15- 2.39)	1.80 (1.20- 3.30)	2.03 (1.36- 5.10)	1.50 (1.20- 2.60)	3.62 (1.49- 17.83)	1.60 (1.10- 3.20)
IL-5 (2.6)	1.49 (1.17- 2.29)	1.54 (1.19- 2.08)	1.60 (1.12- 3.51)	1.55 (1.19- 2.20)	1.60 (1.14- 4.39)	1.74 (1.20- 5.07)	1.46 (1.09- 2.80)
IL-6 (2.6)	2.37 (1.71- 9.87)	17.31 (2.65- 168.0)	1.90 (1.18- 6.14)	12.20 (2.83- 403.7)	2.09 (1.30- 8.37)	156.3 (26.90- 459.4)	1.87 (1.24- 5.30)
IL-8 (3.5)	6.55 (2.38- 15.45)	8.47 (2.33- 51.84)	4.60 (2.69- 10.98)	13.03 (5.79- 59.16)	4.70 (2.63- 10.59)	29.71 (10.12- 152.1)	4.71 (2.85- 11.54)
IL-10 (2.8)	4.50 (2.25- 9.04)	89.19 (9.82- 1,306.0)	4.13 (1.76- 33.67)	64.40 (11.81- 669.1)	4.50 (2.08- 50.22)	533.20 (26.48- 2,415)	4.64 (1.66- 43.99)
IL-12p70 (1.9)	1.52 (1.14- 1.90)	1.55 (1.13- 3.24)	1.85 (1.46- 3.08)	1.65 (1.24- 2.65)	2.13 (1.15- 2.87)	1.84 (1.34- 2.59)	2.09 (1.20- 2.92)
IL-10:TNF-α	3.47 (1.87- 6.31)	43.25 (9.97- 486.0)	2.10 (1.06- 20.20)	25.16 (6.94- 123.5)	2.60 (1.38- 19.54)	140.2 (15.8- 417.4)	3.65 (1.71- 29.83)
IL-10:IL-6	1.63 (0.60- 3.63)	4.58 (1.51- 15.85)	2.20 (0.81- 14.09)	3.92 (1.35- 7.90)	2.20 (0.92- 6.20)	2.93 (0.58- 8.44)	2.20 (0.9- 7.75)
